# Estimation of genetic connectedness diagnostics based on prediction errors without the prediction error variance–covariance matrix

**DOI:** 10.1186/s12711-017-0302-9

**Published:** 2017-03-02

**Authors:** John B. Holmes, Ken G. Dodds, Michael A. Lee

**Affiliations:** 1 Department of Mathematics and Statistics, University of Otago, Cumberland St., Dunedin, 9016 New Zealand; 2 AgResearch, Invermay Research Centre, Puddle Alley, Dunedin, 9053 New Zealand

## Abstract

**Background:**

An important issue in genetic evaluation is the comparability of random effects (breeding values), particularly between pairs of animals in different contemporary groups. This is usually referred to as genetic connectedness. While various measures of connectedness have been proposed in the literature, there is general agreement that the most appropriate measure is some function of the prediction error variance–covariance matrix. However, obtaining the prediction error variance–covariance matrix is computationally demanding for large-scale genetic evaluations. Many alternative statistics have been proposed that avoid the computational cost of obtaining the prediction error variance–covariance matrix, such as counts of genetic links between contemporary groups, gene flow matrices, and functions of the variance–covariance matrix of estimated contemporary group fixed effects.

**Results:**

In this paper, we show that a correction to the variance–covariance matrix of estimated contemporary group fixed effects will produce the exact prediction error variance–covariance matrix averaged by contemporary group for univariate models in the presence of single or multiple fixed effects and one random effect. We demonstrate the correction for a series of models and show that approximations to the prediction error matrix based solely on the variance–covariance matrix of estimated contemporary group fixed effects are inappropriate in certain circumstances.

**Conclusions:**

Our method allows for the calculation of a connectedness measure based on the prediction error variance–covariance matrix by calculating only the variance–covariance matrix of estimated fixed effects. Since the number of fixed effects in genetic evaluation is usually orders of magnitudes smaller than the number of random effect levels, the computational requirements for our method should be reduced.

**Electronic supplementary material:**

The online version of this article (doi:10.1186/s12711-017-0302-9) contains supplementary material, which is available to authorized users.

## Background

A goal of genetic evaluation is to predict genetic merit, while optimising accuracy and minimising bias. Ideally, a breeder of seed stock should be able to compare all individuals in an evaluation irrespective of contemporary group. This is problematic when there is little or no genetic connectedness between groups, unless there is a belief that the model assumptions, specifically assumptions concerning genetic relationships between animals, completely describe the population in question, which is not the case in general. Estimation and reporting of genetic connectedness are important as there are, taking the example of the New Zealand sheep industry, hundreds of flocks evaluated over disparate environments and within each, there are many more contemporary groups. There is sharing of genetic material (rams) between groups and individual seedstock breeders and a centrally co-ordinated progeny test to increase genetic connectedness [1], but many flocks or groups of flocks likely lack genetic connectedness to allow comparison, therefore, in New Zealand, genetic connectedness is reported to seed stock (rams) breeders [2].

In the work of Foulley et al. [3, 4] and Laloë et al. [5, 6], genetic connectedness is regarded as a measure of predictability, where predictability is the random effect extension of estimability [7]. More recently, this was the approach to connectedness taken by Kerr et al. [8]. An estimable function [9, 10] is defined in the context of a fixed effect model. In particular, a function is said to be estimable if vectors $\mathbf{a}$ and $\mathbf{k}$ exist such that $E({\mathbf{a}'y}) = \mathbf{k}' {\varvec{\upbeta }}$. For random effects, all linear combinations can be predicted, regardless of their distribution [3], even if they are not estimable when treated as fixed effects. To get around this, connectedness was defined as the loss of information due to a lack of orthogonality [4] measured by using the Kullback–Leibler divergence. It was shown in Laloë [5] that for a linear mixed model, the expected information is a function of the ratio of the posterior and prior variance for $\mathbf{u}$, alternatively known as the prediction error variance–covariance matrix (PEV) and the relationship matrix, respectively. They also showed that the expected information could be re-arranged to give a co-efficient of determination (CD) statistic [5, 6]. To reduce the computational cost of this measure, simulation and the repeated use of iterative solvers were proposed [11].

Alternative measures of connectedness have been designed either to ease interpretability or minimise computational cost [12–14]. Usually these measures attempt to measure the level of genetic linkage between contemporary groups. They often also allow for the possibility that the model is incorrectly specified, such as omitting genetic groups. They include methods based on PEV, the variance–covariance matrix of estimated fixed effects ${Var}(\hat{{\varvec{\upbeta }}})$, the covariance structure fitted for the random effects (the relationship matrix), or a combination of these. Those based on PEV include the ratio in determinants between full and reduced models [4], differences in PEV of contrasts [12] and correlations of random effect contrasts [15]. Methods based on the variance–covariance matrix of estimated fixed effects include variance of differences between estimated fixed effects (VED) [12], and correlations between estimated fixed effects referred to as connectedness rating (CR) [16]. The fixed effect usually considered is contemporary group (such as flock by year or herd by year). Methods based on the relationship matrix include genetic drift variance [17] and direct genetic links [18, 19].

The focus here is on measures of connectedness that are functions of the PEV or the variance covariance matrix of estimated fixed effects, the links between them and the changes observed as the fitted effect structure is changed. The inclusion of genotype data was also considered to assess the impact changes in the relationship matrix have on the relationship between PEV and the variance–covariance matrix of estimated fixed effects and on the connectedness measure being considered.

The first of the measures that we investigated was the PEV of contemporary group differences (${PEVD}_{ij}$) [12]. This is calculated from $\mathbf{Z}$, the incidence matrix indicating which animals have records, ${\mathbf{x}}_{ij}$, a vector of contrasts comparing two groups *i* and *j* and ${Var}(\hat{\mathbf{u}}-\mathbf{u})$, the prediction error variance–covariance matrix of random effects.

$$\begin{aligned} {PEVD}_{ij} = {\mathbf{x}}_{ij}'{\mathbf{Z}}{Var}(\hat{\mathbf{u}}-{\mathbf{u}}){\mathbf{Z}'}{\mathbf{x}}_{ij}. \end{aligned}$$

If the groups *i* and *j* being compared are contemporary groups such that $\mathbf{x}_{ij}'{} \mathbf{u}$ is the difference in the mean random effect between group *i* and *j*, PEVD can be simplified to a function of the prediction error variance–covariance matrix of random effects averaged by contemporary group.

$$\begin{aligned} {PEVD}_{ij} = \overline{{Var}(\hat{\mathbf{u}}-\mathbf{u})}_{ii}+\overline{{Var}(\hat{\mathbf{u}}-\mathbf{u})}_{jj} -2\overline{{Var}(\hat{\mathbf{u}}-\mathbf{u})}_{ij}. \end{aligned}$$

The coefficient of determination (${CD}({\mathbf{x}}_{ij}$)) [5, 6] is also calculated from ${\mathbf{Z}}, {\mathbf{x}}_{ij}$ and ${Var}(\hat{\mathbf{u}}-\mathbf{u})$ but it also includes ${Var}(\mathbf{u})$.

$$\begin{aligned} {CD}({\mathbf{x}}_{ij})&=\, \frac{{\mathbf{x}}_{ij}'{\mathbf{Z}}{Var}(\hat{\mathbf{u}}){\mathbf{Z}' x}_{ij}}{{\mathbf{x}}_{ij}'{\mathbf{Z}}{Var}({\mathbf{u}}){\mathbf{Z}' x}_{ij}} \\&=\, \frac{{\mathbf{x}}_{ij}'{\mathbf{Z}}({Var}({\mathbf{u}})-{Var}(\hat{\mathbf{u}}-{\mathbf{u}})){\mathbf{Z}' x}_{ij}}{{\mathbf{x}}_{ij}'{\mathbf{Z}}{Var}({\mathbf{u}}){\mathbf{Z}' x}_{ij}}. \end{aligned}$$

Flock correlation (*r*) [15] is calculated from the elements of the prediction error variance–covariance matrix of random effects averaged by contemporary group.

$$\begin{aligned} r_{ij} =\frac{\overline{{Var}(\hat{\mathbf{u}}-\mathbf{u})}_{ij}}{\sqrt{\overline{{Var}(\hat{\mathbf{u}}-\mathbf{u})}_{ii}\overline{{Var}(\hat{\mathbf{u}}-\mathbf{u})}_{jj}}}. \end{aligned}$$

For the variance of differences in management unit effects (VED), Kennedy and Trus [12] used the variances and covariances of estimated contemporary group fixed effects, where $\hat{{\varvec{\upbeta }}}_i$ is the estimated effect for contemporary group *i* and $\hat{{\varvec{\upbeta }}}_j$ is the estimated effect for contemporary group *j*.

$$\begin{aligned} {VED}_{ij} ={Var}(\hat{{\varvec{\upbeta }}})_{ii}+{Var}(\hat{{\varvec{\upbeta }}})_{jj}-2{Var}(\hat{{\varvec{\upbeta }}})_{ij}. \end{aligned}$$

The basis for using VED is that ${Var}(\hat{{\varvec{\upbeta }}})$ is an approximation of $\overline{{Var}(\hat{\mathbf{u}}-\mathbf{u})}$ [12] . In this scenario, VED should estimate the *PEV* of contemporary group differences, PEVD. As a connectedness rating (CR), [16] used the variances and covariances of estimated contemporary group fixed effects, where $\hat{{\varvec{\upbeta }}}_i$ is the estimated effect for contemporary group *i* and $\hat{{\varvec{\upbeta }}}_j$ is the estimated effect for contemporary group *j*.

$$\begin{aligned} { CR}_{ij} =\frac{{Var}(\hat{{\varvec{\upbeta }}})_{ij}}{\sqrt{{Var}(\hat{{\varvec{\upbeta }}})_{ii}{Var}(\hat{{\varvec{\upbeta }}})_{jj}}}. \end{aligned}$$

Using the same argument as for VED, CR approximates the flock correlation. The aim of this paper is to give an exact measure of $\overline{{Var}(\hat{\mathbf{u}}-\mathbf{u})}$ using functions of the variance-covariance matrix of the estimated fixed effects. We also demonstrate that, under certain circumstances, the approximations provide poor estimates of $\overline{{Var}(\hat{\mathbf{u}}-\mathbf{u})}$ and hence are poor predictors of genetic connectedness.

For the remainder of this paper, ${Var}(\hat{\mathbf{u}}-\mathbf{u})$ will be referred to as PEV and $\overline{{Var}(\hat{\mathbf{u}}-\mathbf{u})}$ as PEVMean.

## Materials and methods

### Data

The data available, collected by New Zealand seed stock (ram) breeders and previously used in Holmes et al. [20], consisted of 40,837 animals with live-weight recorded at eight months of age. These animals were born between 2011 and 2013. Together with ancestors, 84,802 animals with pedigree information were obtained from the database of the New Zealand genetic evaluation system for sheep, Sheep Improvement Limited (SIL) [2]. A total of 269 animals were genotyped using the 50K Illumina SNP chip and of these, 21 had live-weight records. A total of 31,615 animals without genotype information were descendants of a genotyped animal. As these data were previously collected by commercial seed stock breeders, special animal ethics authorisation was not required.

### Methods

#### Models

For modelling purposes, we considered the following variables as fixed effects. The contemporary group variable was flock-sex-contemporary group combination, as is standard for growth traits in SIL. There were 202 flock-sex-contemporary groups in the dataset. The combination of birth and rearing rank (four levels) and age of dam (three levels) were treated as categorical variables. Date of birth was treated as a continuous covariate and defined as the difference (in days) between the animals date of birth and the average date of birth in its flock and year combination. Weaning weight was fitted as a continuous covariate. Three models were fitted. Model 1 fitted flock-sex-contemporary group combination as the only fixed effect. Model 2 fitted flock-sex-contemporary group combination, date of birth, and birth rearing rank as fixed effects. Model 3 fitted all available fixed effects. The animal genetic effect was fitted into all models as a random effect. Two variations on the variance-covariance matrix of the random animal effect were considered. These were $\mathbf{A}$ and $\mathbf{H}$. Matrix $\mathbf{A}$ used only the pedigree information available to construct the variance–covariance matrix. The method of Meuwissen and Luo [21] was used to construct the inverse of $\mathbf{A}$ required for the mixed-model equations. Matrix $\mathbf{H}$ used genotype and pedigree information to construct the variance–covariance matrix. The genomic component of the variance–covariance matrix $\mathbf G$ was constructed using the first method of VanRaden [22] and the inverse of $\mathbf{H}$ was constructed using the method outlined in Aguilar et.al. [23]. The variance components were estimated for Model 3 using $\mathbf{A}$ to model the covariance structure of the animal effect in ASReml [24]. Estimates of variance components were $\sigma _g^2 =1.81$ and $\sigma _e^2 = 7.43$ resulting in a heritability of 0.20. Standard errors for the variance components were 0.13 and 0.11 respectively. The variance components were then fixed at these values for all other models, regardless of whether the variance–covariance matrix of the random effect was $\mathbf{A}$ or $\mathbf{H}$.

#### Functions of the fixed effects considered

Three functions of the variance–covariance matrix of estimated fixed effects were compared to the directly calculated PEVMean. Function 1 is the approximation ${Var}(\hat{{\varvec{\upbeta }}}_1)$, where $\hat{{\varvec{\upbeta }}}_1$ is the vector of contemporary group fixed effects. The elements of this function were used to calculate CR [16] and VED [12]. Function 2 is the function of the variance–covariance matrix of estimated fixed effects that gives PEVMean for a model with only one fixed effect fitted.

$$\begin{aligned} PEVMean =\,{Var}(\hat{{\varvec{\upbeta }}}) - \sigma _e^2(\mathbf{X}'{} \mathbf{X})^{-1}. \end{aligned}$$

Function 3 is the function of the variance–covariance matrix of estimated fixed effects that gives PEVMean for a model with multiple fixed effects fitted.

$$\begin{aligned} PEVMean&= {\left( {\mathbf{X}}_1'{\mathbf{X}}_1 \right) ^{-1}{\mathbf{X}}_1'{\mathbf{X}}_2}{Var}(\hat{{\varvec{\upbeta }}}_2){{\mathbf{X}}_2'{} \mathbf{X}_1(\mathbf{X}_1'{} \mathbf{X}_1 )^{-1}} \\&\quad + {(\mathbf{X}_1'{} \mathbf{X}_1 )^{-1}{} \mathbf{X}_1'\mathbf{X}_2}{Var}(\hat{{\varvec{\upbeta }}}_2, \hat{{\varvec{\upbeta }}}_1) \\&\quad + {Var}(\hat{{\varvec{\upbeta }}}_1, \hat{{\varvec{\upbeta }}}_2)\mathbf{X}_2'{} \mathbf{X}_1(\mathbf{X}_1'{} \mathbf{X}_1 )^{-1} \\&\quad + {Var}(\hat{{\varvec{\upbeta }}}_1)- \sigma _e^2(\mathbf{X}_1'{\mathbf{X}}_1 )^{-1}. \end{aligned}$$

The derivations and notations for function 2 and function 3 are in the “Appendix”.

#### Correction factors used in function 2 and function 3

Both function 2 and function 3 are matrix additions to function 1, ${Var}(\hat{{\varvec{\upbeta }}}_1)$. Therefore, the extra calculations required can be regarded as correction factors to obtain PEVMean. In function 2, we subtracted $\sigma _e^2(\mathbf{X}_1'{} \mathbf{X}_1 )^{-1}$ from function 1, where $(\mathbf{X}_1'{} \mathbf{X}_1 )^{-1}$ is a diagonal matrix with entries *ii* equal to $\frac{1}{n_i}$, where $n_i$ is the number of observations in contemporary group *i*. Therefore, $\sigma _e^2(\mathbf{X}_1'{} \mathbf{X}_1 )^{-1}$ is the correction factor for the number of records. Due to the inverse relationship with contemporary group size, this correction is more pronounced for small contemporary groups. Function 3 is the addition of ${(\mathbf{X}_1'{} \mathbf{X}_1 )^{-1}\mathbf{X}_1'{} \mathbf{X}_2}{Var}(\hat{{\varvec{\upbeta }}}_2){\mathbf{X}_2'\mathbf{X}_1(\mathbf{X}_1'{} \mathbf{X}_1 )^{-1}}+ {(\mathbf{X}_1'{} \mathbf{X}_1 )^{-1}\mathbf{X}_1'{} \mathbf{X}_2}{Var}(\hat{{\varvec{\upbeta }}}_2, \hat{{\varvec{\upbeta }}}_1)+ {Var}(\hat{{\varvec{\upbeta }}}_1, \hat{{\varvec{\upbeta }}}_2)\mathbf{X}_2'{} \mathbf{X}_1 (\mathbf{X}_1'{} \mathbf{X}_1 )^{-1}$ to function 2. This addition is therefore the correction to account for the inclusion of other fixed effects in the model.

#### Calculation of connectedness measures and their comparison

The fixed effect variance covariance matrix ${Var}(\hat{{\varvec{\upbeta }}})$ and PEV were extracted from the inverse of the mixed model equations. PEVMean was calculated from PEV. From this, the PEV of contemporary group differences (PEVD) and flock correlation were calculated. From ${Var}(\hat{{\varvec{\upbeta }}}_1)$, VED and CR were calculated. All calculations used R [25].

The three functions described earlier were compared using correlations between the elements of PEVMean and the corresponding elements of the function in question. Diagonal elements were considered separately from off-diagonal elements.

As mentioned in the “Background” section, CR is the analogue to the flock correlation and VED is the analogue to PEVD under the assumption that ${Var}(\hat{{\varvec{\upbeta }}}_1)$ approximates PEVMean. Therefore, correlations between the flock correlation and CR and between VED and PEVD were calculated to assess whether variance of differences or correlation functions of ${Var}(\hat{{\varvec{\upbeta }}}_1)$ gave a more accurate approximation to the corresponding functions of PEVMean than the individual elements of ${Var}(\hat{{\varvec{\upbeta }}}_1)$ did for the individual elements of PEVMean. Both Pearson and Spearman correlations were considered for all examples to assess whether a linear relationship or just the relative rank was maintained.

## Results

### Model 1: Flock-sex-contemporary group interaction is the only fixed effect fitted

Correlations between the elements of PEVMean and the elements of function 1 and function 2 are in Table 1. For function 1, correlations were high for diagonal elements (Pearson: 0.994 for $\mathbf{A}$, 0.994 for $\mathbf{H}$. Spearman: 0.932 for $\mathbf{A}$, 0.928 for $\mathbf{H}$), regardless of whether $\mathbf{A}$ or $\mathbf{H}$ was used as the variance–covariance matrix of the animal random effect. The off-diagonal elements of PEVMean and ${Var}(\hat{{\varvec{\upbeta }}}_1)$ were exactly equivalent. As expected from the derivations earlier, function 2 produced an exact one to one correspondence with PEVMean.

**Table 1:** Pearson and Spearman correlations of PEVMean with functions 1, 2 and 3 for three models and two relationship matrices ($\mathbf{A}$ and $\mathbf{H}$)

Model		Function		
		1	2	3
1 **A**	Diagonals Pearson	0.994	1	NA
	Diagonals Spearman	0.932	1	NA
	Off-diagonal Pearson	1	1	NA
	Off-diagonal Spearman	1	1	NA
1 **H**	Diagonals Pearson	0.994	1	NA
	Diagonals Spearman	0.928	1	NA
	Off-diagonal Pearson	1	1	NA
	Off-diagonal Spearman	1	1	NA
2 **A**	Diagonals Pearson	0.994	1.000*	1
	Diagonals Spearman	0.932	0.999	1
	Off-diagonal Pearson	0.995	0.995	1
	Off-diagonal Spearman	0.625	0.625	1
2 **H**	Diagonals Pearson	0.994	1.000*	1
	Diagonals Spearman	0.928	1.000*	1
	Off-diagonal Pearson	0.996	0.996	1
	Off-diagonal Spearman	0.710	0.710	1
3 **A**	Diagonals Pearson	0.994	1.000*	1
	Diagonals Spearman	0.935	0.980	1
	Off-diagonal Pearson	0.481	0.481	1
	Off-diagonal Spearman	0.423	0.423	1
3 **H**	Diagonals Pearson	0.994	1.000*	1
	Diagonals Spearman	0.931	0.985	1
	Off-diagonal Pearson	0.534	0.534	1
	Off-diagonal Spearman	0.491	0.491	1

Measure 3 is not applicable for Model 1. Correlations marked with a* round to 1 as opposed to being exactly 1

**Fig. 1:**
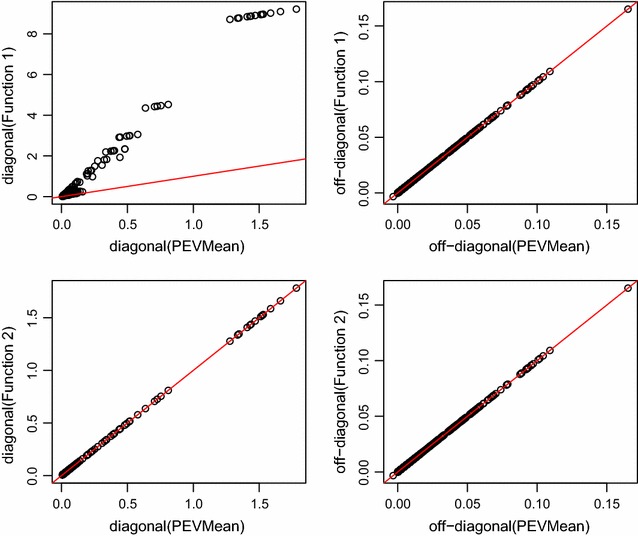
PEVMean against functions 1, and 2 for Model 1 when $\mathbf{A}$ was used. *First column* is diagonal elements, *second column* is off-diagonal elements. The *red line* is equality

A high correlation between the elements of PEVMean and the elements of function 1 and function 2 was observed because the correction to function 1 that is required to obtain PEVMean, when only one fixed effect is fitted, is the correction for the number of records. As mentioned earlier, the correction factor for the number of records was a diagonal matrix and the off-diagonal elements of ${Var}( \hat{{\varvec{\upbeta }}})$ were unchanged when converting to PEVMean. The diagonal elements of PEVMean will be less than ${Var}( \hat{{\varvec{\upbeta }}})$ (Fig. 1), in particular for contemporary groups with few records. This also means that CR consistently gave lower values than the flock correlation.

The basis for using VED was that ${Var}(\hat{{\varvec{\upbeta }}})$ approximated PEVMean. By the same logic, CR should also approximate the flock correlation. Correlations of CR with the flock correlation and of VED with PEVD are in Table 2. Pearson correlations of CR with the flock correlation were lower than the correlation between the elements of function 1 and PEVMean, which are in Table 1. Spearman correlations of CR with the flock correlation were higher. Correlations between VED and PEVD were high, but Pearson correlations were higher than Spearman correlations. This was as expected based on the high correlations for both the diagonals and off-diagonals. However, the values of VED were in a higher range than PEVD due to the inflation of diagonal elements of ${Var}( \hat{{\varvec{\upbeta }}})$ compared to PEVMean. The inflation of VED compared to PEVD, due to not applying the correction factor for the number of records, was most pronounced for small contemporary groups.

**Table 2:** Pearson and Spearman correlations of the flock correlation with CR and PEVD with VED for three models and two relationship matrices ($\mathbf{A}$ and $\mathbf{H}$)

Model	Correlation type	Flock correlation against CR	PEVD against VED
1 **A**	Pearson	0.943	0.994
	Spearman	0.999	0.942
1 **H**	Pearson	0.945	0.994
	Spearman	0.999	0.938
2 **A**	Pearson	0.914	0.994
	Spearman	0.534	0.942
2 **H**	Pearson	0.927	0.994
	Spearman	0.636	0.938
3 **A**	Pearson	0.430	0.994
	Spearman	0.258	0.939
3 **H**	Pearson	0.481	0.994
	Spearman	0.345	0.934

### Model 2: Contemporary group, date of birth and birth rearing rank fitted

Correlations between the elements of PEVMean and the elements of function 1, function 2 and function 3 are in Table 1. Correlations between the elements of PEVMean and function 1 were high for diagonal elements but lower for off-diagonal elements. Due to the inclusion of non-contemporary group fixed effects, elements of function 2 did not give an exact correspondence to the elements of PEVMean. In function 2, correlations with the diagonal elements of PEVMean increased compared to function 1, while the off-diagonal elements were unchanged because the correction factor for the number of records applied to diagonals only. As expected from the derivations obtained above, function 3 produced an exact one to one correspondence with PEVMean.

**Fig. 2:**
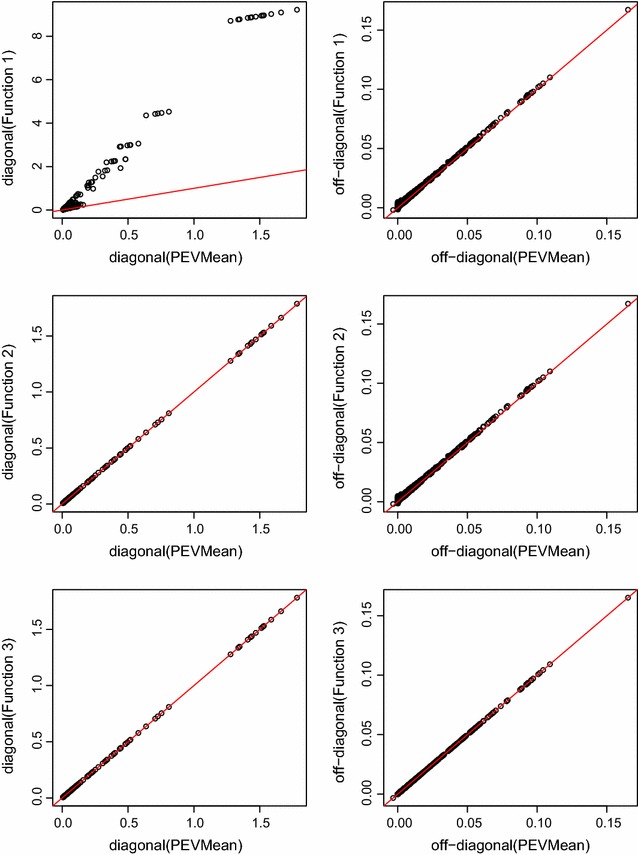
PEVMean against functions 1, 2, and 3 for Model 2 when $\mathbf{A}$ was used. *First column* is diagonal elements, *second column* is off-diagonal elements. The *red line* is equality

The diagonal elements of function 2 gave almost a one to one correspondence with the diagonal elements of PEVMean regardless of whether $\mathbf{A}$ (Fig. 2) or $\mathbf{H}$ was used. This indicates that the magnitude of the correction factor to account for the other fixed effects was negligible relative to the magnitude of function 2. The correction factor lowered the off-diagonal elements of ${Var}(\hat{{\varvec{\upbeta }}}_1)$ uniformly. For both diagonal and off-diagonal elements, the relative impact of including the correction for other fixed effects in the model was therefore higher for elements with a lower absolute value.

Inclusion of other fixed effects lowered the correlation between CR and the flock correlation and between VED and PEVD compared to model 1. CR usually gave lower values than the flock correlation. Exceptions were due to both the diagonal and off-diagonal elements of ${Var}(\hat{{\varvec{\upbeta }}}_1)$ that overestimated the corresponding element of PEVMean. Correlations between VED and PEVD were high; with Pearson correlations higher than Spearman correlations. As in Model 1, VED had a higher range than PEVD.

### Model 3: Contemporary group, age of dam, date of birth, birth rearing rank and flock $\times$ sex interaction fitted

Correlations between the elements of PEVMean and function 1 were high for diagonal elements but lower for off-diagonal elements. Inclusion of additional fixed effects means that, as in Model 2, elements of function 2 did not give an exact correspondence to the elements of PEVMean. Correlations of the diagonal elements of function 2 with the diagonal elements of PEVMean increased compared to function 1, while the off-diagonal elements were unchanged because the correction factor for the number of records applies to diagonals only. As expected from the derivations obtained above, function 3 produced an exact one to one correspondence with PEVMean.

**Fig. 3:**
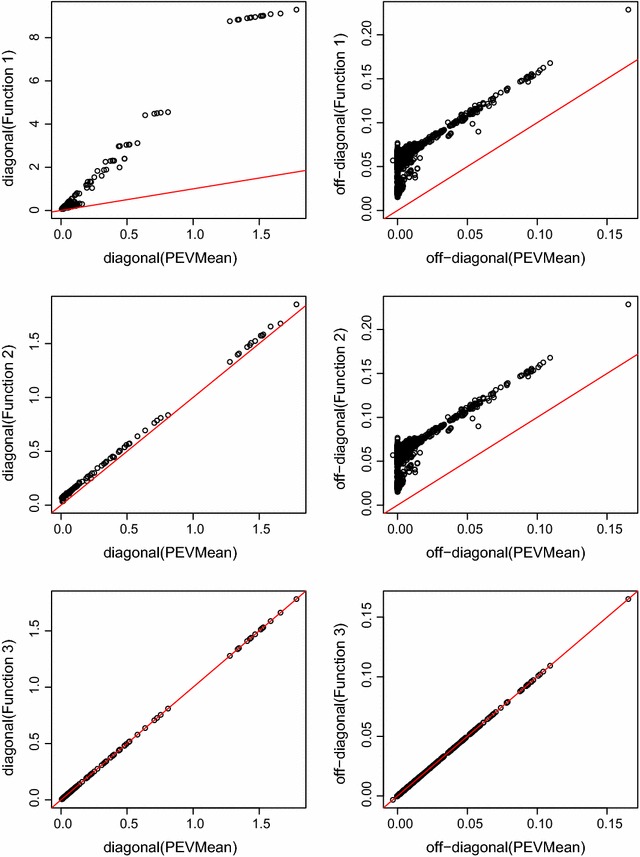
PEVMean against functions 1, 2, and 3 for Model 3 when $\mathbf{A}$ was used. *First column* is diagonal elements, *second column* is off-diagonal elements. The *red line* is the 45 degree *line*

The correction factor to account for the other fixed effects in the model was typically about 35 times larger than in Model 2. As a result, diagonal elements of function 2 were increased compared to diagonal elements of PEVMean (Fig. 3). For the off-diagonal elements, the correction factor accounting for other fixed effects in the model was uniform when the off-diagonal element of PEVMean moved away from zero. There was more variation in the correction factor when the off-diagonal element of PEVMean was near zero. Inflation seen in off-diagonal elements of function 1 compared to off-diagonal elements of PEVMean was due primarily to not correcting for other fixed effects rather than not correcting for the number of records. CR generally gave larger estimates than the flock correlation and over-estimation was most pronounced when off-diagonal elements of PEVMean and hence the flock correlation were near zero.

Inclusion of weaning weight and age of dam in the model decreased the correlations of CR with the flock correlation compared to Models 1 and 2 (Table 2). In particular, flock correlations that approach 0 in this model may have a high CR. The reasons for this will be elaborated in the “Discussion” section. The largest difference between CR and the flock correlation was between contemporary groups 98 and 107 when $\mathbf{A}$ was used (flock correlation = 0.022, CR = 0.818), and between contemporary groups 147 and 152 when $\mathbf{H}$ was used (flock correlation = 0.056, CR = 0.803). The correlation between VED and PEVD remained high in Model 3.

### Impact of using $\mathbf{H}$ compared to $\mathbf{A}$ to model the variance–covariance of the animal random effect

The use of $\mathbf{H}$ instead of $\mathbf{A}$ did not significantly change the Pearson correlation of PEVMean with the approximations functions 1 and 2, except for the off-diagonals in Model 3 (Table 1). Similarly, it did not result in large differences in the Pearson correlations between CR and the flock correlation or between VED and PEVD, except between CR and the flock correlation in Model 3 (Table 2). The use of $\mathbf{H}$ increased the Spearman correlations for off-diagonal elements of PEVMean with functions 1 and 2 (Table 1) and of CR with the flock correlation (Table 2) for Models 2 and 3.

Additional file 1: Figure S1 shows the impact of using $\mathbf{H}$ as opposed to $\mathbf{A}$, which was to increase PEVMean, particularly when the value of PEVMean using $\mathbf{A}$ was near zero. This was particularly obvious for the off-diagonals. The result was an increase in the flock correlation and CR compared to the equivalent model in which $\mathbf{A}$ was fitted.

### Patterns in the correction factor accounting for the inclusion of other fixed effects in the model

**Fig. 4:**
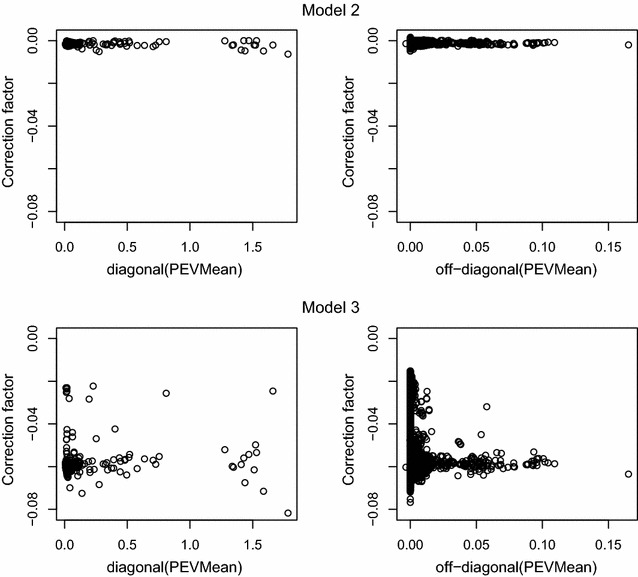
PEVMean against correction factor for Model 2 and Model 3 when $\mathbf{A}$ was used

The relationship between the correction factor and the PEVMean for the two models (Models 2 and 3), for which the correction factor was relevant is in Fig. 4. The correction factor was similar for both the diagonal and off-diagonal elements. There was no relationship between the value of the correction factor and the value of PEVMean, except for an increase in variability in the correction factor when the element of PEVMean was near zero. The correction factor was approximately 35 times larger in Model 3 than in Model 2, as indicated by traces of the correction factor. The low degree of variation in the correction factor for other fixed effects suggested that the dataset that we used was approximately balanced across contemporary groups.

### Patterns in connectedness rating (CR) and variance of estimated differences of management units (VED)

#### Connectedness rating

**Fig. 5:**
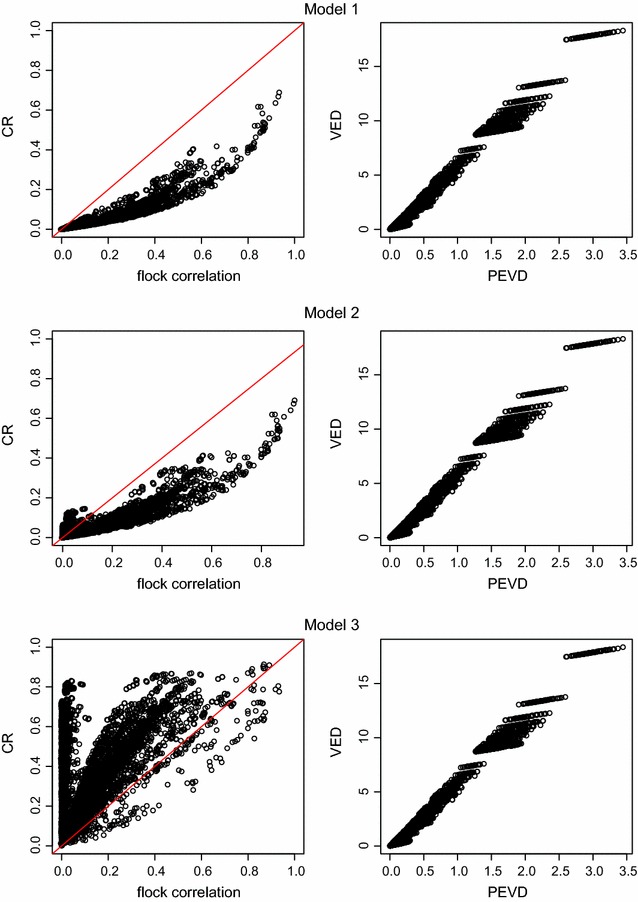
Flock correlation against CR and PEVD against VED when $\mathbf{A}$ was used. The *first column* is Flock correlation against CR. The *second column* is PEVD against VED. The *red line* in *first column* is equality

The flock correlation was compared to CR (Fig. 5). As mentioned, CR underestimated the flock correlation in Model 1 for all pairs of contemporary groups and for most pairs in Model 2. Conversely, CR overestimated the flock correlation for most pairs in Model 3. In Model 2 and especially in Model 3, there was a collection of contemporary group pairs for which the flock correlation was near zero (completely disconnected), while the corresponding CR estimate was much higher than zero. This was due to the correction factor for the other fitted fixed effects, which was similar for both the diagonal and off-diagonal elements, and had the largest impact on very small covariances and hence correlations. The divergence between CR and the flock correlation when the flock correlation was near zero was also a function of contemporary group size. Since the variances were inversely dependent on the number of records in the contemporary group, the most pronounced differences between CR and flock correlation occurred between contemporary groups that were not linked and had a large number of records. Additional file 2: Figure S2 shows the relationship between the harmonic mean $\frac{2}{\frac{1}{n_1}+\frac{1}{n_2}}$ and CR when the corresponding flock correlation is low. For Model 2 and especially Model 3, higher harmonic means were associated with higher CR.

#### Variance of estimated differences of management units (VED)

Unlike CR compared to the flock correlation, VED showed a stronger relationship with PEVD (Fig. 5). However, for all three models, there were certain pairs of contemporary groups that had similar VED, but substantially different PEVD. This variation increased PEVD and was probably due to VED not correcting for the number of records in each contemporary group because VED, PEVD and the correction factor for the number of records were all inversely dependent on the number of records in the contemporary groups in question. Table 3 shows that VED corrected for the number of records was equivalent to PEVD in Model 1, as expected, while the corrected VED showed a near one to one relationship with PEVD for both Models 2 and 3. An almost exact one to one relationship between corrected VED and PEVD for Models 2 and 3 was due to the correction factor for the other fixed effects being fairly uniform and thus cancelling out in the calculation of variances of differences, which both VED and PEVD are examples of.

**Table 3:** Simple linear regression between VED corrected for the number of records and PEVD for three models and two relationship matrices ($\mathbf{A}$ and $\mathbf{H}$)

Model	Intercept	Slope	$r^2$
1, **A**	0	1	1
1, **H**	0	1	1
2, **A**	0.000*	1.001	1.000*
2, **H**	0.000*	1.001	1.000*
3, **A**	0.004	1.002	1.000*
3, **H**	0.004	1.002	1.000*

Numbers with a * only round to and are not exactly 0 or 1

## Discussion

### Sensitivity to the presence of other fixed effects in the model fitted

In the example used by Kennedy and Trus [12], a correlation of 0.995 was found between ${Var}(\hat{{\varvec{\upbeta }}}_1)$ and the mean PEV. However, they only considered a model where contemporary group was the only fixed effect. For the three models that we fitted, the correlation between the variance–covariance matrix of estimated contemporary group fixed effects and the prediction error variance–covariance matrix of contemporary group averages was sensitive to the inclusion of other fixed effects in the model. This sensitivity depended on the correction factor for the other fixed effects included in the model.

#### Situations where it is unnecessary to use the correction factor for other fixed effects included in the model

If we assume that the incidence matrices for the contemporary group effect $\mathbf{X}_1$ and the other fixed effects $\mathbf{X}_2$ are orthogonal, then $\mathbf{X}_1'{} \mathbf{X}_2 = \mathbf{0}$. In this scenario, the correction factor for the other fixed effects included in the model becomes zero and the calculation of PEVMean from the variance–covariance matrix of estimated fixed effects can be done as if the contemporary group is the only fixed effect. An individual element *ij* of matrix $\mathbf{X}_1'{} \mathbf{X}_2$ represents the number of observations of effect *j* in contemporary group level *i* if the other effect is a factor and is the sum of the covariate values for effect *j* in the contemporary level *i* if effect *j* is continuous. In practice, $\mathbf{X}_1'{} \mathbf{X}_2 = \mathbf{0}$ would be limited to the situation where the other fixed effects considered in the model are continuous, centred on zero and balanced across all levels of the contemporary group effect, i.e. the mean of the other variables is zero for all contemporary group levels.

#### Situations where parts of the correction factor for the other fixed effects in the model can be ignored

If all the columns of the other fixed effects present in the model lie in the null-space of ${\mathbf{X}_1'}{Var}(\mathbf y)^{-1}$, where $\mathbf{X}_1$ is the incidence matrix of contemporary group effects and ${Var}(\mathbf{y}) = {\mathbf{Z}}{Var}(\mathbf{u}){\mathbf{Z}'} + \sigma ^2_e \mathbf I$ is the variance–covariance matrix of the observations, then ${Var}(\hat{{\varvec{\upbeta }}}_2, \hat{{\varvec{\upbeta }}}_1) = 0$ and the correction factor for the other fitted fixed effects reduces to ${(\mathbf{X}_1'{} \mathbf{X}_1 )^{-1}{} \mathbf{X}_1'{} \mathbf{X}_2}{Var}(\hat{{\varvec{\upbeta }}}_2) \mathbf{X}_2'{} \mathbf{X}_1(\mathbf{X}_1'{} \mathbf{X}_1 )^{-1}$. The variance–covariance matrix of estimated contemporary group effects ${Var}(\hat{{\varvec{\upbeta }}}_1)$ is unchanged when moving from the reduced model, (only contemporary group is fitted) compared to a full model where other fixed effects are fitted. To measure how close the model considered could come to such a state, the covariance ratio [26] was considered. The covariance ratio is the ratio of determinants for ${Var}(\hat{{\varvec{\upbeta }}})$ between a full and reduced model. Therefore, it is similar to the $\gamma$ statistic proposed by Foulley et al. [3]. In our particular case, we considered the covariance ratio of contemporary group effects between a full and reduced model. If the correction factor reduced to ${(\mathbf{X}_1'{} \mathbf{X}_1 )^{-1}{} \mathbf{X}_1'\mathbf{X}_2}{Var}(\hat{{\varvec{\upbeta }}}_2)\mathbf{X}_2'{} \mathbf{X}_1(\mathbf{X}_1'{} \mathbf{X}_1 )^{-1}$, the covariance ratio was equal to 1. A covariance ratio that diverged from 1 indicates that estimates of ${Var}(\hat{{\varvec{\upbeta }}})_1$ are influenced by the addition of more fixed effects. The covariance ratios of the three models fitted are in Table 4. The covariance ratio for Model 1 compared to Model 2 (0.406 when $\mathbf{A}$ was used and 0.452 when $\mathbf{H}$ was used) is close to one, while for Model 1 compared to Model 3, it was not (0.005 when $\mathbf{A}$ was used, 0.006 when $\mathbf{H}$ was used).

**Table 4:** Covariance ratio for the variance–covariance matrix of estimated contemporary group fixed effects for three models and two relationship matrices ($\mathbf{A}$ and $\mathbf{H}$)

	$${Var}(\hat{{\varvec{\upbeta }}})$$		
	Model 1	Model 2	Model 3
$$\mathbf{A}$$			
$${Var}(\hat{{\varvec{\upbeta }}})^{-1}$$			
Model 1	1	2.462	210.041
Model 2	0.406	1	85.239
Model 3	0.005	0.001	1
$$\mathbf{H}$$			
$${Var}(\hat{{\varvec{\upbeta }}})^{-1}$$			
Model 1	1	2.213	166.744
Model 2	0.452	1	75.354
Model 3	0.006	0.013	1

Covariance ratio is defined as $\det ({Var}(\hat{{\varvec{\upbeta }}})_{\text {A}} {Var}(\hat{{\varvec{\upbeta }}})_{\text {B}}^{-1})$ where *A* and *B* represent nested models. The model indicated in the column heading is *A*, the model in the row heading is *B*

#### Correction factor for other fixed effects in the model when those effects are balanced across contemporary groups.

When all other effects in the model are balanced across contemporary group, defined as having equal means (if continuous) or occurring for the same proportion of observations (if factors) for all contemporary groups, then the elements in each row of the incidence matrix $(\mathbf{X}_1'{} \mathbf{X}_1 )^{-1}{} \mathbf{X}_1'{} \mathbf{X}_2$ are the same. Therefore, $(\mathbf{X}_1'{} \mathbf{X}_1 )^{-1}{} \mathbf{X}_1'{} \mathbf{X}_2 = \mathbf{1}{} \mathbf{r}'$, where $\mathbf{1}$ and $\mathbf{r}$ are column vectors of length $p_1$ and $p_2$, respectively, and $p_1, p_2$ are the number of contemporary group and non-contemporary group effect levels in the model. As a consequence, ${(\mathbf{X}_1'{} \mathbf{X}_1 )^{-1}{} \mathbf{X}_1'{} \mathbf{X}_2}{Var}(\hat{{\varvec{\upbeta }}}_2){\mathbf{X}_2'{} \mathbf{X}_1(\mathbf{X}_1'{} \mathbf{X}_1 )^{-1}} = {\mathbf{1}{} \mathbf{r}'} {Var}(\hat{{\varvec{\upbeta }}}_2) {\mathbf{r} \mathbf{1}'} = {\mathbf{r}'} {Var}(\hat{{\varvec{\upbeta }}}_2) \mathbf{r}{} \mathbf{1} \mathbf{1}' = c \mathbf{1}{} \mathbf{1}'$, where *c* is the constant ${\mathbf{r}'} {Var}(\hat{{\varvec{\upbeta }}}_2) \mathbf{r}$ and $\mathbf{1} \mathbf{1}'$ a $p_1 \times p_1$ matrix of ones. In this situation, the relationship between VED and PEVD simplifies to the result below when contemporary group is the only fixed effect fitted.

$$\begin{aligned} { PEVD}_{ij}=\,&{} {Var}(\hat{{\varvec{\upbeta }}}_1)_{ii} + {Var}(\hat{{\varvec{\upbeta }}}_1)_{jj}-2{Var}(\hat{{\varvec{\upbeta }}}_1)_{ij} \\&+c{(\mathbf{1}{} \mathbf{1}')}_{ii} +({\mathbf{1}{} \mathbf{r}'}{Var}(\hat{{\varvec{\upbeta }}}_2, \hat{{\varvec{\upbeta }}}_1))_{ii} \\&+c{(\mathbf{1}{} \mathbf{1}')}_{jj}+({\mathbf{1}{} \mathbf{r}'}{Var}(\hat{{\varvec{\upbeta }}}_2, \hat{{\varvec{\upbeta }}}_1))_{jj} \\&-2c{(\mathbf{1}{} \mathbf{1}')}_{ij}-2({\mathbf{1}{} \mathbf{r}'}{Var}(\hat{{\varvec{\upbeta }}}_2, \hat{{\varvec{\upbeta }}}_1))_{ij} \\&+ ({Var}\left( \hat{{\varvec{\upbeta }}}_1, \hat{{\varvec{\upbeta }}}_2\right) {\mathbf{r} \mathbf{1}'})_{ii} \\&+({Var}(\hat{{\varvec{\upbeta }}}_1, \hat{{\varvec{\upbeta }}}_2){\mathbf{r} \mathbf{1}'})_{jj} \\&-2({Var}(\hat{{\varvec{\upbeta }}}_1, \hat{{\varvec{\upbeta }}}_2){\mathbf{r} \mathbf{1}'})_{ij} \\&-\sigma _e^2({\mathbf{X}_1'{} \mathbf{X}_1})^{-1}_{ii} -\sigma _e^2({\mathbf{X}_1'{} \mathbf{X}_1})^{-1}_{jj} \\= & {} {Var}(\hat{{\varvec{\upbeta }}}_1)_{ii} + {Var}(\hat{{\varvec{\upbeta }}}_1)_{jj}-2{Var}(\hat{{\varvec{\upbeta }}}_1)_{ij} \\&+{Var}({\mathbf{r}'}\hat{{\varvec{\upbeta }}}_2, \hat{{\varvec{\upbeta }}}_1)_{i} + {Var}({\mathbf{r}'}\hat{ {\varvec{\upbeta }}}_2, \hat{{\varvec{\upbeta }}}_1)_{j} \\&-2{Var}({\mathbf{r}'} \hat{{\varvec{\upbeta }}}_2, \hat{{\varvec{\upbeta }}}_1)_{j}+{Var}({\mathbf{r}'}\hat{ {\varvec{\upbeta }}}_2, \hat{{\varvec{\upbeta }}}_1)_{i} \\&+ {Var}({\mathbf{r}'} \hat{{\varvec{\upbeta }}}_2, \hat{{\varvec{\upbeta }}}_1)_{j} -2{Var}( {\mathbf{r}'}\hat{{\varvec{\upbeta }}}_2, \hat{{\varvec{\upbeta }}}_1)_{i} \\&-\sigma _e^2({\mathbf{X}_1'{} \mathbf{X}_1})^{-1}_{ii} -\sigma _e^2({\mathbf{X}_1'{} \mathbf{X}_1})^{-1}_{jj} \\=\,& {} {Var}(\hat{{\varvec{\upbeta }}}_1)_{ii} + {Var}(\hat{{\varvec{\upbeta }}}_1)_{jj}-2{Var}(\hat{{\varvec{\upbeta }}}_1)_{ij} \\&-\sigma _e^2({\mathbf{X}_1'{} \mathbf{X}_1})^{-1}_{ii}-\sigma _e^2({\mathbf{X}_1'{} \mathbf{X}_1})^{-1}_{jj}\\= & {} {VED}_{ij}-\sigma _e^2({\mathbf{X}_1'\mathbf{X}_1})^{-1}_{ii}-\sigma _e^2({\mathbf{X}_1'{} \mathbf{X}_1})^{-1}_{jj} \end{aligned}$$

#### Sensitivity to the mean of continuous covariates fitted in the model

To obtain the relationship between PEVMean and the variance–covariance matrix of estimated contemporary group fixed effects, the intercept must be absorbed into the contemporary group effects. The variance of the intercept depends on the mean of the variables included in the model [9]. By absorbing the intercept into the contemporary groups, ${Var}(\hat{{\varvec{\upbeta }}}_1)$ becomes dependent on the means of the other variables included in the model. Since PEVMean itself is invariant to rescaling of continuous fixed effects, the impact of the correction factor for the other fixed effects in the model is itself influenced by the means of the other effects. This can be illustrated by fitting a fourth model. Model 4 is equivalent to Model 3 except that the weaning weight covariate is standardised to have a mean of 0 and standard deviation of 1. The zero mean for weaning weight minimises the influence of the weaning weight covariate on ${Var}(\hat{{\varvec{\upbeta }}}_1)$. While the PEVMean was unchanged when moving from Model 3 to Model 4, Additional file 3: Figure S3 shows that the correction factor for the other fixed effects in the model was reduced. It also reduced but did not eliminate the overestimation of flock correlation when using CR, particularly when the flock correlation was near zero.

#### Link to postulated mixed model $r^2$ and correction factor for the inclusion of other fixed effects

To measure the impact of including fixed effects other than contemporary group into the model, we considered the coefficient of determination ($r^2$). Unlike the general linear model, linear mixed models do not have a commonly agreed $r^2$ statistic. We considered two methods to measure $r^2_m$ for the fixed effect component of the model. The first was marginal $r^2$ [27]. This was calculated as:

$$\begin{aligned} r^2_{m}=\frac{Var( \hat{y})}{Var(\hat{y})+\sigma _e^2+\sigma _g^2}, \end{aligned}$$

where $\hat{y}$ were the predicted values for the observation without the random effects. The second method was $r^2_\upbeta$ [28]. This is calculated as a function of the Wald F statistic, $\hat{{\varvec{\upbeta }}}' \mathbf {V}(\hat{{\varvec{\upbeta }}})^{-1}\hat{{\varvec{\upbeta }}}$ with $n-p$ as $\nu$, where *n* was the number of observations, and *p* was the number of fixed effects to be estimated.

$$\begin{aligned} r^2_\upbeta = \frac{(q-1)F(\hat{{\varvec{\upbeta }}},Var(\mathbf{Y})) }{\nu +(q-1)F(\hat{{\varvec{\upbeta }}},Var(\mathbf{Y})) }. \end{aligned}$$

While we did find the $r^2$ statistics useful for indicating improvement in model fit, we did not find any relationship with the correction factor. Therefore, $r^2$ statistics like those considered should not be used as a diagnostic of the impact that the inclusion of additional fixed effects in the model had on the correction factor.

### A diagnostic to assess the need to include the correction factor

The value of the correction factor for calculating PEVMean from ${Var}(\hat{{\varvec{\upbeta }}})$ can be assessed as the trace of the matrix of the correction factor for other fixed effects included in the model. Specifically, the trace was considered as a diagnostic to determine whether it is appropriate to just use ${Var}(\hat{{\varvec{\upbeta }}}_1) - (\mathbf{X}'\mathbf{X})^{-1}\sigma _e^2$ as an approximation to PEVMean. The trace of the correction factor can be written as $2Tr({Var}(\hat{{\varvec{\upbeta }}}_2, \hat{{\varvec{\upbeta }}}_1)({\mathbf{X}_1'{} \mathbf{X}_1 )^{-1}\mathbf{X}_1'{} \mathbf{X}_2})+Tr({Var}(\hat{{\varvec{\upbeta }}}_2)\mathbf{X}_2'{} \mathbf{X}_1 (\mathbf{X}_1'{} \mathbf{X}_1)^{-1}(\mathbf{X}_1'{} \mathbf{X}_1 )^{-1}{} \mathbf{X}_1'{} \mathbf{X}_2 )$. This formulation was less computationally demanding when the number of contemporary group fixed effect levels was greater than the number of other fixed effect levels. Traces that were further from zero indicated that the correction factor had a greater impact in the calculation of PEVMean. Table 5 provides the traces of the correction factor for the inclusion of other fixed effects in the model. For Model 3 the trace is approximately 35 times greater than for Model 2, which suggests that ignoring the other fixed effects in Model 3 results in a poor approximation of PEVMean.

**Table 5:** Trace of the correction factor for the inclusion of additional fixed effects

Model	$\mathbf{A}$	$\mathbf{H}$
2	−0.3310	−0.3298
3	−11.4795	−11.5005

### Utility of the method

#### Solving blocks of the mixed model equations

The exact PEVMean given by function 3 requires the calculation of the variance–covariance matrix for all estimated fixed effects in the model. This can be done directly by calculating $({\mathbf{X}'}{Var}(\mathbf y)^{-1}{} \mathbf{X})^{-1}$, where ${Var}(\mathbf{y}) = {\mathbf{Z} }{Var}({\mathbf{u}}) {\mathbf{Z}}' + \sigma ^2_e\mathbf{I}$. This is computationally demanding since the direct inversion of $Var(\mathbf y)$ requires $n^2(n+1)/2$ operations, where *n* is the number of observations. An alternative method is to find the block of the mixed model equation inverse corresponding to the fixed effects. Mathur et al. [16] wrote a program that calculated these blocks for CR. Many software programs have in-built functions that can be used to solve equations of the form $\mathbf{A} \mathbf{X} = \mathbf{B}$, where $\mathbf{A}$ and $\mathbf{B}$ are known matrices. Examples include the solve() function in R [25]. Using this method to find ${Var}(\hat{{\varvec{\upbeta }}})$, $\mathbf{A}$ would be the mixed model equation matrix and $\mathbf{B}$ would be the first *p* columns of the identity matrix, where *p* is the number of fixed effects to be estimated in the model. The elements of PEVMean can then be calculated from ${Var}(\hat{{\varvec{\upbeta }}})$. However this method would also calculate ${Var}(\hat{{\varvec{\upbeta }}},\hat{\mathbf{u}}-\mathbf{u})$ in addition to ${Var}(\hat{{\varvec{\upbeta }}})$.

#### Calculating PEVMean from ${Var}(\hat{{\varvec{\upbeta }}})$

After ${Var}(\hat{{\varvec{\upbeta }}})$ is obtained, the number of operations required to obtain the components that go into function 3 is as follows. To avoid re-calculation of the same matrix, we assume that these steps are done in the order outlined in Table 6. Since $\mathbf{X}'{} \mathbf{X}$ is required to form the mixed model equations, $\mathbf{X}'_1\mathbf{X}_2$ is assumed to have no cost. In the number of operations, $p_1$ represents the number of contemporary group fixed effects and $p_2$ represents the number of other fixed effects estimated.

**Table 6:** Operations required to calculate the correction factor

Step	Component	Number of operations
1	$(\mathbf{X}_1' \mathbf{X}_1)^{-1}$	$p_1$
2	${\mathbf{X}_1'{} \mathbf{X}_2}{Var}(\hat{{\varvec{\upbeta }}}_2){\mathbf{X}_2'{} \mathbf{X}_1}$	$p_1p_2^2+p_1^2p_2$
3	Multiplying $(\mathbf{X}_1'{} \mathbf{X}_1 )^{-1}$ on both sides of step 2	$2p_1^2$
4	${\mathbf{X}_1'{} \mathbf{X}_2}{Var}(\hat{{\varvec{\upbeta }}}_2, \hat{{\varvec{\upbeta }}}_1)$	$p_1^2p_2$
5	Multiplying $(\mathbf{X}_1'{} \mathbf{X}_1 )^{-1}$ on the left side of step 4	$p_1^2$
6	Addition to obtain correction factor for other fixed effects	$2p_1^2$
7	Addition of step 6 to ${Var}(\hat{{\varvec{\upbeta }}}_1)$	$p_1^2$
8	Completing *PEVMean*	$p_1$
	Total calculations	$2p_1+6p_{1}^{2} +p_1p_2(2p_1+p_2)$

In the models we considered $p_1>> p_2$. This means that the number of operations required to obtain *PEVMean* after ${Var}(\hat{{\varvec{\upbeta }}})$ was obtained is of order $p_1^2$.

## Conclusions

For single-trait models in which only one random effect is fitted, a function of the variance-covariance matrix of all fixed effects fitted can be used to calculate the prediction error variance-covariance matrix averaged by contemporary group. Depending on the other fixed effects included, the use of just the elements of the variance–covariance matrix of the estimated contemporary group fixed effects can give suboptimal estimates of connectedness. This is particularly the case when correlation-based measures are used, such as CR. These inaccuracies can be reduced by centring any continuous variables included in the model to have a mean of zero. When difference-based measures such as PEVD are used, the need to consider the other fitted fixed effects is eliminated when those effects are balanced across the contemporary groups effect levels. Nevertheless, there was always a notable improvement in the approximation of PEVMean by subtracting $\sigma _e^2(\mathbf{X}_1' \mathbf{X}_1)^{-1}$ from ${Var}(\hat{{\varvec{\upbeta }}}_1)$.

The proposed formula for calculating PEVMean from ${Var}(\hat{{\varvec{\upbeta }}})$ can be also used to calculate the flock correlation, the prediction error variance of differences, and the PEV component of the coefficient of determination for contrasts between contemporary groups by calculating only the block of the inverse of the mixed model equations corresponding to the fixed effects, rather than the full prediction-error variance–covariance matrix of random effects. By being able to calculate PEVMean exactly from functions of ${Var}(\hat{{\varvec{\upbeta }}})$, a more accurate assessment of connectedness can be obtained in livestock genetic evaluation compared to traditional fixed effect based measures such as connectedness rating and VED, without the computational cost of PEV based measures. A future goal of research is to give tractable solutions to calculate this for industry evaluations which may include millions of animals. In addition, tens of thousands of these animals will typically have genotype data and in the future this number will increase and hence will require a re-evaluation of the connectedness measures used in the New Zealand sheep industry. Better measures of genetic connectedness between groups will allow seed stock breeders to make better decisions on the appropriateness of comparing animals in evaluations, which will, in an industry such as the New Zealand sheep industry, lead to increased genetic gain.

## Appendix

### Derivation of the *PEVMean* as a function of the variance–covariance matrix of estimated fixed effects only

The equation of a linear mixed model has the following matrix form:

$$\begin{aligned} \mathbf{y = \mathbf{X} {\varvec{\upbeta }} + \mathbf{Z}{} \mathbf{u}+\mathbf e} ,{Var}({\mathbf{u}}) =\mathbf{G}, {Var}(\mathbf e)=\mathbf{R}. \end{aligned}$$

Solutions for ${\varvec{\upbeta }}, \mathbf{u}$ can be found by solving the mixed model equations derived by Henderson [29]:

1 $$\mathbf{X}' {\mathbf{R}}^{-1}{} \mathbf{X}\hat{{\varvec{\upbeta }}} + \mathbf{X' \mathbf{R}}^{-1}{} \mathbf{Z}\hat{\mathbf{u}}=\mathbf{X'{} \mathbf{R}}^{-1}\mathbf{y}$$

2 $${\mathbf{Z}}'{\mathbf{R}}^{-1}{} \mathbf{X}\hat{{\varvec{\upbeta }}} + ({\mathbf{Z}'{} \mathbf{R}}^{-1}{\mathbf{Z}}+\mathbf{G}^{-1})\hat{\mathbf{u}}={\mathbf{Z}'{} \mathbf{R}}^{-1}\mathbf{y}.$$

The exact relationship between *PEV* and the variance of estimated fixed effects ${Var}(\hat{{\varvec{\upbeta }}})$ was found by taking the variance on both sides of Eq. (1) and applying the results for the variances from mixed model equations [10].

3 $$\begin{aligned} {Var}(\mathbf{X'{} \mathbf{R}}^{-1}{} \mathbf{X}\hat{{\varvec{\upbeta }}} + \mathbf{X'{} \mathbf{R}}^{-1}{} \mathbf{Z}\hat{\mathbf{u}}) &= {Var}(\mathbf{X'\mathbf{R}}^{-1}\mathbf y) \nonumber \\ {Var}({\mathbf{X'{} \mathbf{R}}^{-1}\mathbf{X}\hat{{\varvec{\upbeta }}}}) + {Var}(\mathbf{X'{} \mathbf{R}}^{-1}\mathbf{Z}\hat{\mathbf{u}}) &= {Var}(\mathbf{X'{} \mathbf{R}}^{-1}\mathbf y) \nonumber \\ {\mathbf{X'{} \mathbf{R}}^{-1}}({\mathbf{X}}{Var}(\hat{{\varvec{\upbeta }}}){\mathbf{X}'} + {\mathbf{Z}}{Var}(\hat{\mathbf{u}}){\mathbf{Z}'}){\mathbf{R}}^{-1}{} \mathbf{X} &= {\mathbf{X'{} \mathbf{R}}^{-1}}({ Var}({\mathbf{Z}{} \mathbf{u}})+{Var}(\mathbf e)){\mathbf{R}}^{-1}{} \mathbf{X} \nonumber \\ {\mathbf{X'{} \mathbf{R}}^{-1}}({\mathbf{X}}{Var}(\hat{{\varvec{\upbeta }}}){\mathbf{X}'} + {\mathbf{Z}}{Var}(\hat{\mathbf{u}}){\mathbf{Z}'}){\mathbf{R}}^{-1}{} \mathbf{X} &= {\mathbf{X'{} \mathbf{R}}^{-1}}({\mathbf{Z}}{Var}(\mathbf{u})\mathbf{Z}'+\mathbf{R}){\mathbf{R}}^{-1}{} \mathbf{X} \nonumber \\ {\mathbf{X'{} \mathbf{R}}^{-1}(\mathbf{X}}{Var}(\hat{{\varvec{\upbeta }}}){\mathbf{X}' -{\mathbf{R}}){\mathbf{R}}^{-1}{} \mathbf{X}} &= {\mathbf{X'{} \mathbf{R}}^{-1}(\mathbf{Z}}{Var}({\mathbf{u}}){\mathbf{Z}'}- {\mathbf{Z}}{Var}(\hat{\mathbf{u}})\mathbf{Z}')\mathbf{R}^{-1}{} \mathbf{X} \nonumber \\ {\mathbf{X'{} \mathbf{R}}^{-1}(\mathbf{X}}{Var}(\hat{{\varvec{\upbeta }}}){\mathbf{X}' -{\mathbf{R}}){\mathbf{R}}^{-1}{} \mathbf{X}} &= {\mathbf{X'{} \mathbf{R}}^{-1}{} \mathbf{Z}}({Var}({\mathbf{u}})-{Var}(\hat{\mathbf{u}}))\mathbf{Z}'{} \mathbf{R}^{-1}{} \mathbf{X} \nonumber \\ {\mathbf{X'{} \mathbf{R}}^{-1}\mathbf{X}}{Var}(\hat{{\varvec{\upbeta }}}){\mathbf{X'{} \mathbf{R}}^{-1}{} \mathbf{X} -\mathbf{X'{} \mathbf{R}}^{-1}{} \mathbf{X}} &= {\mathbf{X'{} \mathbf{R}}^{-1}\mathbf{Z}}{Var}(\hat{\mathbf{u}}-{\mathbf{u}}){\mathbf{Z}'{} \mathbf{R}}^{-1}{} \mathbf{X} \end{aligned}$$

To simplify the result, we assumed that ${\mathbf{R}} = \sigma _e^2\mathbf{I}$ where $\mathbf I$ is the identity matrix. This simplified the result in Eq. (3) to:

4 $$\begin{aligned} {\mathbf{X}'{} \mathbf{X}}{Var}(\hat{{\varvec{\upbeta }}}){\mathbf{X}'\mathbf{X}} - \sigma _e^2{\mathbf{X}'{} \mathbf{X}}= & {} {\mathbf{X}'{} \mathbf{Z}}{Var}(\hat{\mathbf{u}}-\mathbf{u})\mathbf{Z}'{} \mathbf{X} \nonumber \\ {Var}({\mathbf{X}'{} \mathbf{X}}\hat{{\varvec{\upbeta }}})- \sigma _e^2{\mathbf{X}'{} \mathbf{X}}= & {} {{Var}(\mathbf{X}'{} \mathbf{Z}}(\hat{\mathbf{u}}-\mathbf{u})). \end{aligned}$$

For the derivations of function 2 and function 3, it was assumed that the intercept was absorbed into the contemporary group fixed effect.

#### Formula for function 2

If contemporary group is the only fixed effect included, $\mathbf{X}'{} \mathbf{X}$ is a diagonal matrix with the entry $({\mathbf{X'\mathbf X}})_{ii}$ corresponding to the number of observations in contemporary group *i*. The entries of $\mathbf{X}'{} \mathbf{Z}$ are an incidence matrix indicating which contemporary group a particular animal belongs to. In this setting, the matrix $(\mathbf{X}'\mathbf{X})^{-1}{} \mathbf{X}'{} \mathbf{Z}$ is the linear transformation from $\mathbf{u}$ to $\bar{\mathbf{u}}$, where $\bar{\mathbf{u}}$ is the vector of breeding values averaged by contemporary group. This simplifies Eq. 4 as follows.

$$\begin{aligned} {(\mathbf{X}'{} \mathbf{X})^{-1}}{Var}(\mathbf{X'{} \mathbf{Z}}(\hat{\mathbf{u}}-{\mathbf{u}})) (\mathbf{X}'{} \mathbf{X})^{-1}=\,& {} {(\mathbf{X}'\mathbf{X})^{-1}}({Var}({\mathbf{X}'{} \mathbf{X}}\hat{{\varvec{\upbeta }}}) \\&- \sigma _e^2\mathbf{X}'{} \mathbf{X})(\mathbf{X}'{} \mathbf{X})^{-1} \\ {Var}((\mathbf{X}'{} \mathbf{X})^{-1}{} \mathbf{X}'{} \mathbf{Z}(\hat{\mathbf{u}}-\mathbf{u}))=\,& {} {Var}(\hat{{\varvec{\upbeta }}}) - \sigma _e^2(\mathbf{X}'\mathbf{X})^{-1} \\ {Var}(\overline{\hat{\mathbf{u}}-\mathbf{u}})=\,& {} {Var}(\hat{{\varvec{\upbeta }}}) - \sigma _e^2(\mathbf{X}'\mathbf{X})^{-1} \end{aligned}$$

Thus, in this scenario *PEVMean* $= {Var}(\hat{{\varvec{\upbeta }}}) - \sigma _e^2(\mathbf{X}'\mathbf{X})^{-1}$ as shown above.

#### Formula for function 3

If contemporary group is not the only fixed effect included, the incidence matrix, $\mathbf{X}$, is split into two parts. $\mathbf{X}_1$ is the incidence matrix for contemporary groups and $\mathbf{X}_2$ is the incidence matrix for other contemporary groups. In this setting, the matrix $(\mathbf{X}_1'{} \mathbf{X}_1)^{-1}{} \mathbf{X}_1'{} \mathbf{Z}$ is the linear transformation from *u* to $\bar{u}$ with respect to contemporary groups. To derive function 3, Eq. 4 was re-written partitioning $\mathbf{X}$ as described and similarly partitioning $\hat{{\varvec{\upbeta }}}$ into $\hat{{\varvec{\upbeta }}}_1, \hat{{\varvec{\upbeta }}}_2$, which are the vectors of estimated contemporary group and non-contemporary group fixed effects respectively.

5 $$\begin{aligned} &\left(\begin{array}{c}{\mathbf{X}}_1'\mathbf{Z}\\ \mathbf{X}_2'{}\mathbf{Z} \end{array}\right){\textit{Var}}({\hat{\mathbf{u}}}-\mathbf{u})\left(\begin{array}{cc} {\mathbf{Z}}' \mathbf{X}_1&{\mathbf{Z}'}{} \mathbf{X}_2\end{array}\right) \\ &\quad=\left(\begin{array}{cc} \mathbf{X}_1'{} \mathbf{X}_1 & \mathbf{X}_1'{} \mathbf{X}_2\\ \mathbf{X}_2'{} \mathbf{X}_1 & \mathbf{X}_2'{} \mathbf{X}_2 \end{array}\right) \left(\begin{array}{cc} {\textit{Var}}(\hat{{\varvec{\upbeta}}}_1)&{\textit{Var}}(\hat{{\varvec{\upbeta}}}_1,\hat{{\varvec{\upbeta}}}_2)\\ {\textit{Var}}(\hat{{\varvec{\upbeta}}}_2,\hat{{\varvec{\upbeta}}}_1) &{\textit{Var}}(\hat{{\varvec{\upbeta}}}_2) \end{array}\right)\\ &\quad\times\left(\begin{array}{cc} \mathbf{X}_1'{}\mathbf{X}_1 &\mathbf{X}_1'{} \mathbf{X}_2\\ \mathbf{X}_2'{}\mathbf{X}_1 &\mathbf{X}_2'{} \mathbf{X}_2 \end{array}\right) - \sigma _e^2 \left(\begin{array}{cc} \mathbf{X}_1'{} \mathbf{X}_1 &\mathbf{X}_1'\mathbf{X}_2\\ \mathbf{X}_2'{} \mathbf{X}_1 &\mathbf{X}_2'{} \mathbf{X}_2 \end{array}\right). \end{aligned}$$

To complete the derivation, the top left block of Equation 5 which corresponds to ${Var}(\mathbf{X}'_1\mathbf{Z}(\hat{\mathbf{u}}-\mathbf{u}))$ was re-arranged.

$$\begin{aligned} {Var}(\mathbf{X}'_1\mathbf{Z}(\hat{\mathbf{u}}-\mathbf{u}))=\,& {} {\mathbf{X}_1'\mathbf{X}_1}{Var}(\hat{{\varvec{\upbeta }}}_1){\mathbf{X}_1'{} \mathbf{X}_1 + \mathbf{X}_1'{} \mathbf{X}_2}{Var}(\hat{{\varvec{\upbeta }}}_2)\mathbf{X}_2'{} \mathbf{X}_1\\&+ {\mathbf{X}_1'{} \mathbf{X}_2}{Var}(\hat{{\varvec{\upbeta }}}_2, \hat{{\varvec{\upbeta }}}_1){\mathbf{X}_1'{} \mathbf{X}_1} + {\mathbf{X}_1'{} \mathbf{X}_1}{Var}(\hat{{\varvec{\upbeta }}}_1, \hat{{\varvec{\upbeta }}}_2)\mathbf{X}_2'{} \mathbf{X}_1 - \sigma _e^2\mathbf{X}'_1\mathbf{X}_1\\ {Var}((\mathbf{X}_1'{} \mathbf{X}_1 )^{-1}{} \mathbf{X}'_1\mathbf{Z}(\hat{\mathbf{u}}-\mathbf{u}))=\,& {} {(\mathbf{X}_1'\mathbf{X}_1 )^{-1}{} \mathbf{X}_1'\mathbf{X}_1}{Var}(\hat{{\varvec{\upbeta }}}_1)\mathbf{X}_1'{} \mathbf{X}_1(\mathbf{X}_1'{} \mathbf{X}_1 )^{-1}\\&+ {(\mathbf{X}_1'{} \mathbf{X}_1 )^{-1}{} \mathbf{X}_1'\mathbf{X}_2}{Var}(\hat{{\varvec{\upbeta }}}_2, \hat{{\varvec{\upbeta }}}_1)\mathbf{X}_1'{} \mathbf{X}_1(\mathbf{X}_1'\mathbf{X}_1 )^{-1} \\&+ {(\mathbf{X}_1'{} \mathbf{X}_1 )^{-1}{} \mathbf{X}_1'\mathbf{X}_1}{Var}(\hat{{\varvec{\upbeta }}}_1, \hat{{\varvec{\upbeta }}}_2)\mathbf{X}_2'{} \mathbf{X}_1(\mathbf{X}_1'\mathbf{X}_1 )^{-1}\\&+ {(\mathbf{X}_1'{} \mathbf{X}_1 )^{-1}{} \mathbf{X}_1'\mathbf{X}_2}{Var}(\hat{{\varvec{\upbeta }}}_2)\mathbf{X}_2'{} \mathbf{X}_1(\mathbf{X}_1'{} \mathbf{X}_1 )^{-1} \\&- \sigma _e^2(\mathbf{X}_1'{} \mathbf{X}_1 )^{-1}{} \mathbf{X}'_1\mathbf{X}_1(\mathbf{X}_1'{} \mathbf{X}_1 )^{-1}\\ {Var}(\overline{\hat{\mathbf{u}}-\mathbf{u}})=\,& {} {Var}(\hat{{\varvec{\upbeta }}}_1)+{ (\mathbf{X}_1'{} \mathbf{X}_1 )^{-1}{} \mathbf{X}_1'{} \mathbf{X}_2}{Var}(\hat{{\varvec{\upbeta }}}_2)\mathbf{X}_2'{} \mathbf{X}_1(\mathbf{X}_1'{} \mathbf{X}_1 )^{-1} \\&+ {(\mathbf{X}_1'{} \mathbf{X}_1 )^{-1}{} \mathbf{X}_1'\mathbf{X}_2}{Var}(\hat{{\varvec{\upbeta }}}_2, \hat{{\varvec{\upbeta }}}_1) \\&+ {Var}(\hat{{\varvec{\upbeta }}}_1, \hat{{\varvec{\upbeta }}}_2)\mathbf{X}_2'{} \mathbf{X}_1(\mathbf{X}_1'\mathbf{X}_1 )^{-1}- \sigma _e^2(\mathbf{X}_1'{} \mathbf{X}_1 )^{-1} \end{aligned}$$

Thus in this scenario, *PEVMean* $={Var}(\hat{{\varvec{\upbeta }}}_1) + (\mathbf{X}_1'{} \mathbf{X}_1 )^{-1} {\mathbf{X}_1'\mathbf{X}_2}{Var}(\hat{{\varvec{\upbeta }}}_2){\mathbf{X}_2'{} \mathbf{X}_1(\mathbf{X}_1'{} \mathbf{X}_1 )^{-1}}+ {(\mathbf{X}_1'{} \mathbf{X}_1 )^{-1}{} \mathbf{X}_1'\mathbf{X}_2} {Var}(\hat{{\varvec{\upbeta }}}_2, \hat{{\varvec{\upbeta }}}_1) + {Var}(\hat{{\varvec{\upbeta }}}_1, \hat{{\varvec{\upbeta }}}_2)\mathbf{X}_2'{} \mathbf{X}_1(\mathbf{X}_1'\mathbf{X}_1 )^{-1} - \sigma _e^2(\mathbf{X}_1'{} \mathbf{X}_1 )^{-1}$ as shown above.

## Additional files

**Additional file 1: Figure S1.** A pdf file containing figures showing the differences in PEVMean between when **H** as opposed to **A** was used to model $Var({{\bf u}})$. First column is diagonal elements, second column is off-diagonal elements. The red line indicates where the element of PEVMean was equal if either **H** and **A** was used.

**Additional file 1: Figure S2.** A pdf file containing figures showing the relationship between flock harmonic mean and the CR when flock correlation is below 0.01 and **A** was used. The left hand side is model 2, the right side is model 3.

**Additional file 1: Figure S3.** A pdf file containing figures showing the relationship of the correction factor and CR between Models 3 and 4 when **A** was used. The first column is Correction factor. The second column is CR. The red line on second column is equality.
