# Supplementary figures and images for: Estimation of genetic connectedness diagnostics based on prediction errors without the prediction error variance–covariance matrix
Source: Genet Sel Evol. 2017 Mar 2;49:29. doi: 10.1186/s12711-017-0302-9 (PMC5439142; doi:10.1186/s12711-017-0302-9)

diagonal(PEVMean)

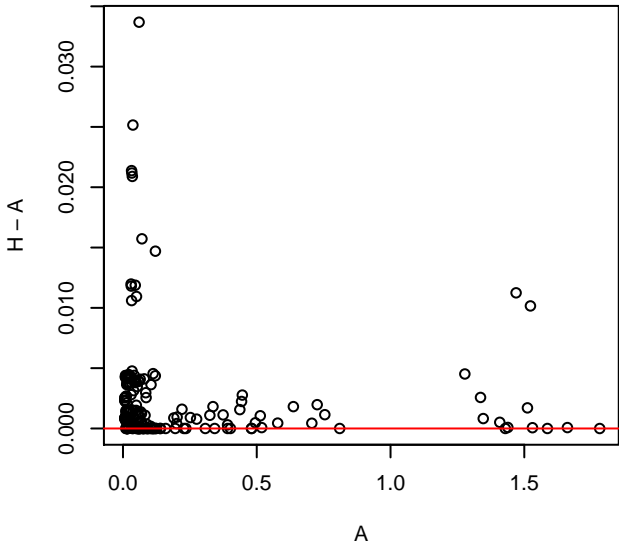

Model 1

off-diagonal(PEVMean)

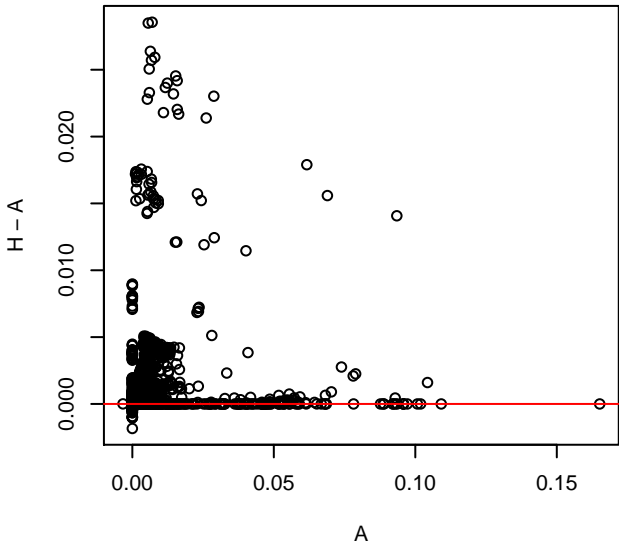

Model 2

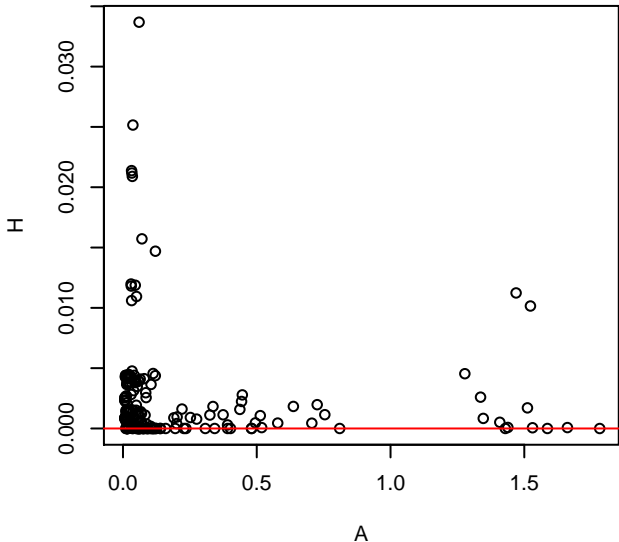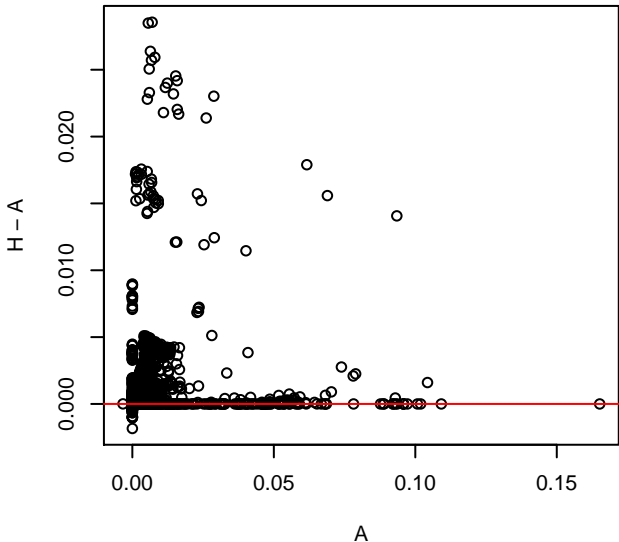

Model 3

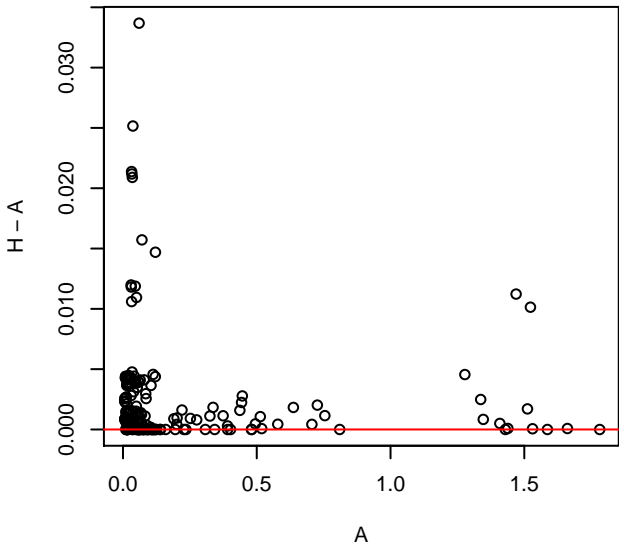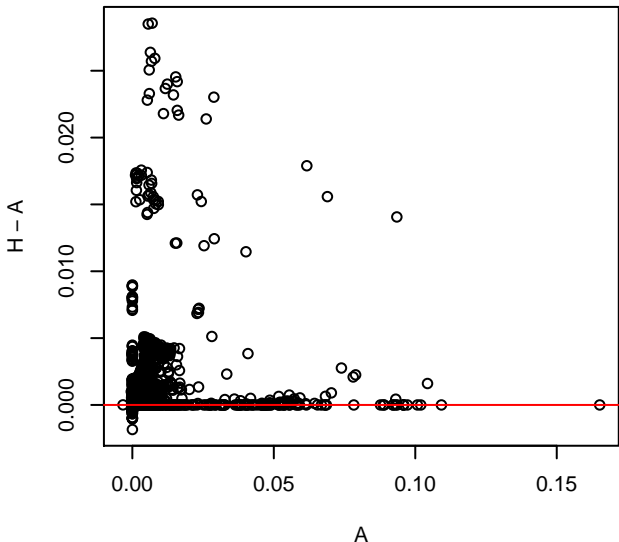

Supplement: Supplementary file 1 — Additional file 1: Figure S1. A pdf file containing figures showing the differences in PEVMean between when H as opposed to A was used to model $Var({{\bf u}})$Var(u). First column is diagonal elements, second column is off-diagonal elements. The red line indicates where the element of PEVMean was equal if either H and A was used. [file 12711_2017_302_MOESM1_ESM.pdf]

**Model 2**

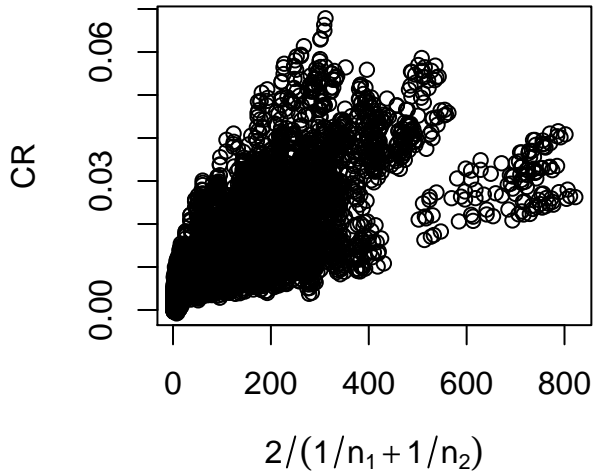

**Model 3**

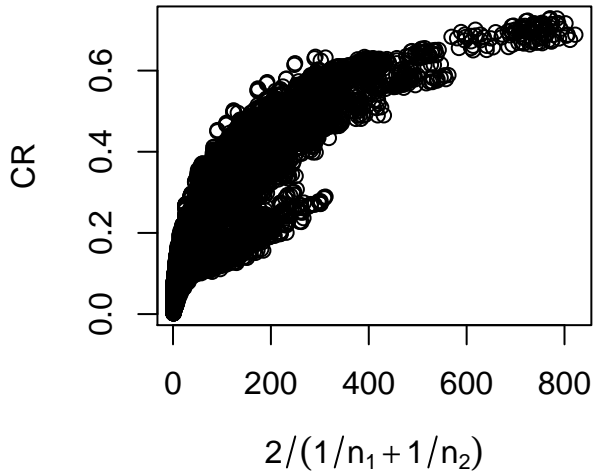

Supplement: Supplementary file 2 — Additional file 1: Figure S2. A pdf file containing figures showing the relationship between flock harmonic mean and the CR when flock correlation is below 0.01 and A was used. The left hand side is model 2, the right side is model 3. [file 12711_2017_302_MOESM2_ESM.pdf]

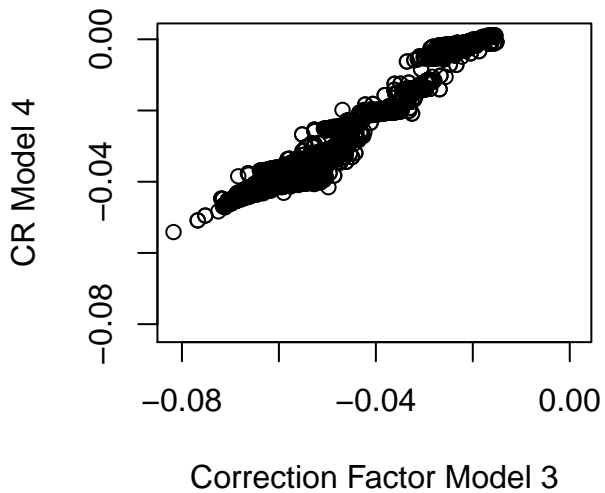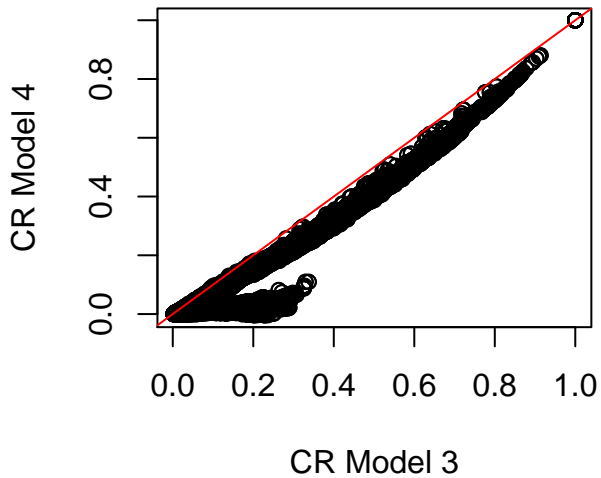

Supplement: Supplementary file 3 — Additional file 1: Figure S3. A pdf file containing figures showing the relationship of the correction factor and CR between Models 3 and 4 when A was used. The first column is Correction factor. The second column is CR. The red line on second column is equality. [file 12711_2017_302_MOESM3_ESM.pdf]
